# Congenital Anomalies in Infant With Congenital Hypothyroidism: A Review of Pathogenesis, Diagnostic Options, and Management Protocols

**DOI:** 10.7759/cureus.24669

**Published:** 2022-05-02

**Authors:** Kivonika Uthayaseelan, Monika Kadari, Muhammad Subhan, Nisha Saji Parel, Parimi Vamsi Krishna, Anuradha Gupta, Kamsika Uthayaseelan

**Affiliations:** 1 Internal Medicine, All Saints University School of Medicine, Roseau, DMA; 2 Internal Medicine, Bhaskar Medical College, Hyderabad, IND; 3 Jinnah Hospital, Allama Iqbal Medical College, Lahore, PAK; 4 Family Medicine, Tbilisi State Medical University, Tbilisi, GEO; 5 Internal Medicine, Jagadguru Jayadeva Murugarajendra (JJM) Medical College, Davanagere, IND; 6 Research, Government Medical College, Nagpur, IND; 7 Internal Medicine, All Saints University College of Medicine, Saint Vincent and the Grenadines, Kingstown, VCT

**Keywords:** primary hypothyroidism, levothyroxine, thyroid, thyroid dysgenesis, extrathyroidal, congenital malformations, pediatric hypothyroidism, hypothyroidism, congenital hypothyroidism, cretinism

## Abstract

Thyroid hormones (TH) regulate growth, nervous system myelination, metabolism, and physiologic functions in nearly every organ system. Congenital hypothyroidism (CH) is one of the most common endocrinopathies in children and has potentially devastating neurologic and developmental consequences. The etiology and clinical manifestations of hypothyroidism in children differ from adults. And hence, pediatric medical care requires a detailed understanding of thyroid function and dysfunction in children. The perinatal risk factors include female sex, preterm birth, low birth weight, postmature birth, additional birth abnormalities, and being delivered in multiple births. In countries where newborn screening is practiced, CH is detected after birth through screening tests. It aids in determining the underlying cause, though some patients may be able to start treatment without these tests. Early detection and treatment prevent irreversible and permanent nervous system damage. Thus, in addition to exploring the development of CH, this article has also covered the epidemiological data, clinical aspects, and management stemming from pediatric hypothyroidism.

## Introduction and background

Thyroid hormones (TH) are necessary for energy metabolism, body temperature regulation, growth, bone production, and central nervous system maturation and are essential for proper growth and brain development in infants [1-2]. Hypothyroidism is defined as a low level of TH in the bloodstream and leads to insufficient metabolic and neurologic effects at the cellular level [1]. Our understanding of the physiological background of pediatric hypothyroidism has improved significantly during the last 20 years [3]. Fisher et al. (1964) discovered that just a tiny amount of thyroxine (T4) crosses through the placental barrier, leaving the fetus dependent on its thyroid gland [3]. About 85% of cases of congenital hypothyroidism (CH) are sporadic while 15% are genetic (autosomal recessive) [4]. In the United States of America (USA), more than four million infants are checked each year, and 1,000 infants are diagnosed with hypothyroidism [4]. According to one study, one in 2,000 Hispanic infants, one in 4,000 white infants, and one in 32,000 African American infants were affected [4]. CH is common in twins, and nearly all screening reports that females have a higher rate of hypothyroidism than males, approaching a 2:1 female/male ratio [4-5]. CH has been related to maternal perinatal factors like advanced maternal age and gestational difficulties and neonatal-perinatal factors like female sex, preterm birth, low birth weight, postmature birth, other birth abnormalities, and being born as part of multiple births [6]. CH is categorized into transient and permanent forms [7]. Transient CH refers to temporary TH deficiency detected at birth but resolved within the first several months or years of life, and permanent CH is a TH deficiency that needs lifetime treatment [5]. Screening programs are first accomplished to reduce the neurodevelopmental consequences of late-treated CH [8]. They have been a significant success, with most primary CH cases diagnosed due to newborn screening. Most countries worldwide use a thyroid-stimulating hormone (TSH)-based screening technique in patients with primary CH, in which TSH is first measured [8]. It is usually on a filter paper blood sample, and only newborns with an elevated TSH are tested for free thyroxine (FT4) or T4 [8]. In contrast, central CH can only be detected by programs measuring T4 or FT4 at the beginning or concurrently with TSH [8]. TH replacement, both the duration and the dose, has been believed to affect neurological outcomes in studies [9]. Hypothyroidism has profound consequences on cardiovascular, neurologic, gastrointestinal, and metabolic function because the thyroid hormone regulates nearly every organ system [10]. Developmental and growth disorders are nearly twice as much in patients with CH compared to normal individuals. This high risk of developmental problems significantly impairs regular activity and standard of living. In this review article, we aim to explore the development of CH along with their pathogenesis, epidemiology, diagnosis, management, and prevention methods.

## Review

Development of congenital hypothyroidism

**Figure 1:**
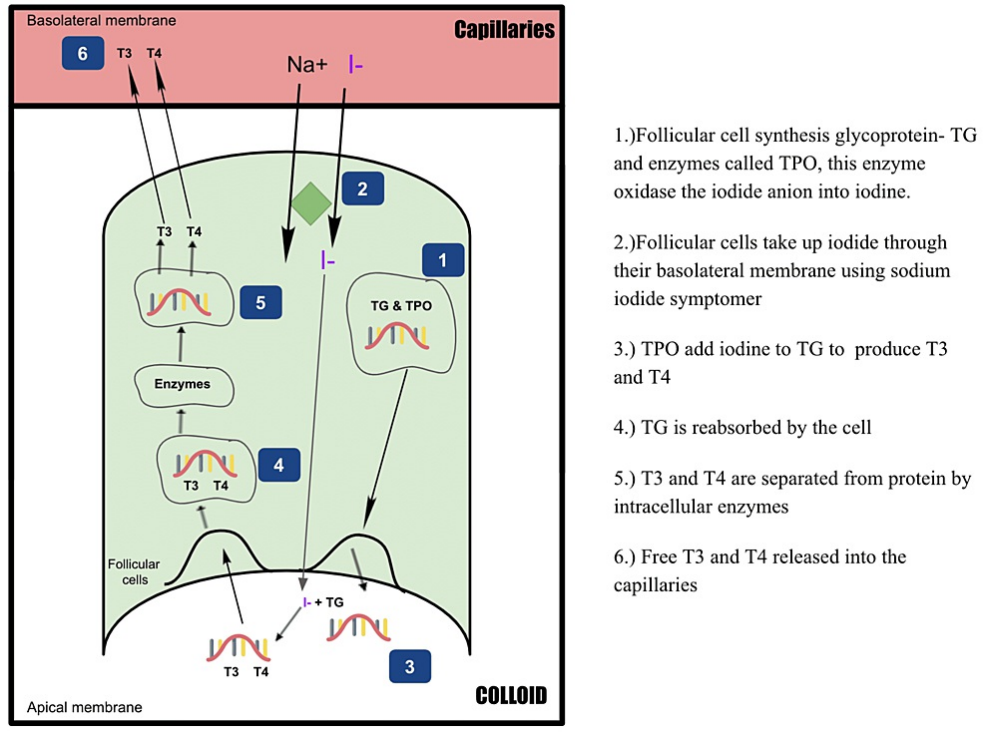
Thyroid hormone synthesis TG: thyroglobulin; TPO: thyroid peroxidase; I-: iodide; Na+: sodium; T3: triiodothyronine; T4: thyroxine Image credits: Kivonika Uthayaseelan

Maturation of thyroid function involves organogenesis, the development of the hypothalamus, pituitary, and thyroid gland, and the growth of TH synthesis and their actions [11]. The thyroid gland is the first endocrine gland to emerge from the endoderm at the foramen cecum around day 24 of gestation [11]. It derives from medial out-pouching from the primitive pharyngeal floor and the fourth pharyngeal pouch [12]. By seven weeks of pregnancy, the tiny butterfly-shaped gland is visible at the base of the neck, consisting of two lobes joined by a thin isthmus tissue and composed of cells known as thyroid follicles [13]. T4 and triiodothyronine (T3) are the primary hormones produced by the thyroid gland [14]. Thyroglobulin (TG) synthesis is found after four weeks, iodine trapping after seven to nine weeks, and T4 production after 12 weeks [15]. The synthesis of T3 and T4 is summarized in Figure 1.

Regulation of congenital hypothyroidism

**Figure 2:**
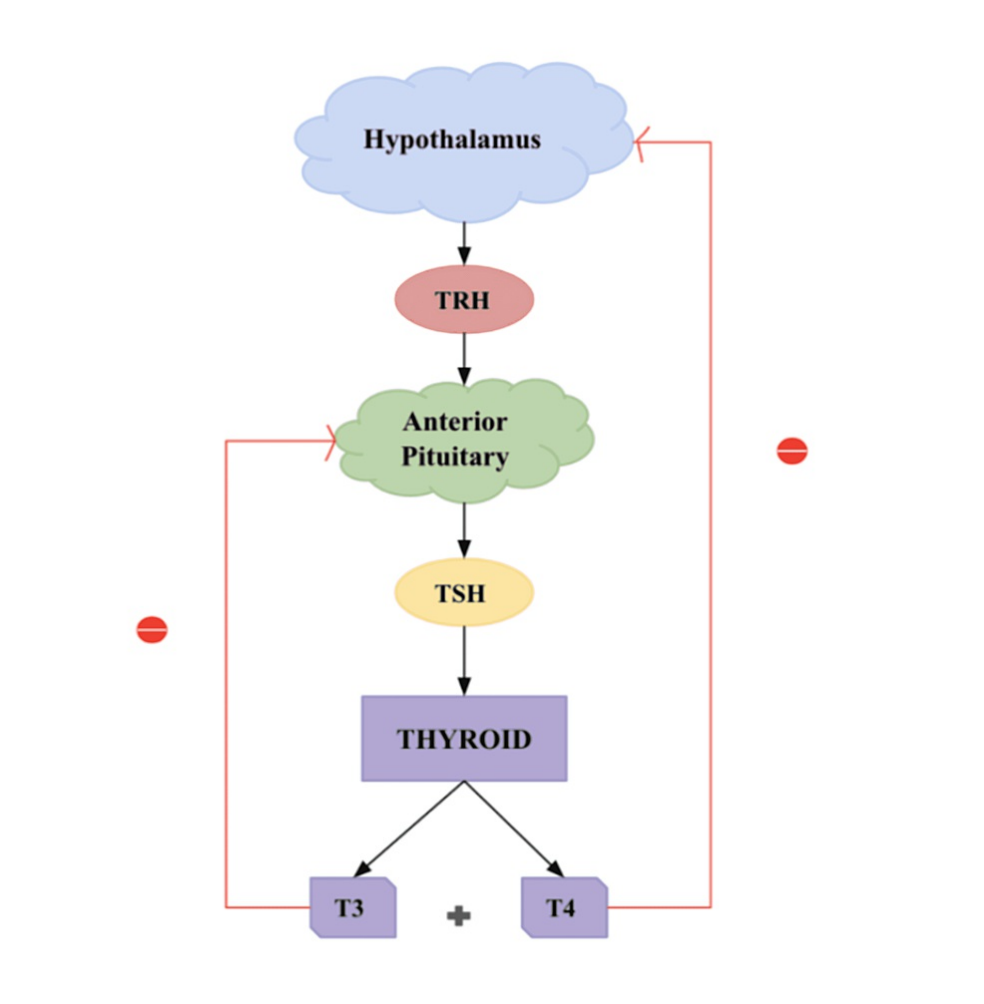
Hypothalamic-pituitary thyroid axis TRH: thyrotropin-releasing hormone; TSH: thyroid-stimulating hormone; T3: triiodothyronine; T4: thyroxine Image credits: Kivonika Uthayaseelan

The anterior pituitary gland and hypothalamus regulate thyrotropin-releasing hormone (TRH) [16]. TRH stimulates TSH to secrete T3 and T4 [16]. By six to eight weeks of gestation, TRH is present in hypothalamus neurons, and TSH secretion can be measured by 12 weeks [17]. When circulating levels of T3 and T4 are low, TRH and TSH induce the release of T3 and T4 into the bloodstream [16]. Because T3 has a short half-life compared to T4, around 80% of this will be T4 and only 10% will be active T3 [10]. T4 and T3 TH are essential for fetal growth and development, but the fetal thyroid gland does not produce significant amounts until the second trimester [18]. Therefore, the fetus depends on the mother to contribute as the primary source of TH in pregnancy, especially in the first trimester [18]. Indeed, fetal T4 levels in the first trimester reflect maternal TH levels, and this dependency decreases as fetal TH production increases and the hypothalamic-pituitary-thyroid (HPT) axis matures [18]. However, the mother's T4 includes 30% to 50% of T4 measured in cord blood at birth, demonstrating that the fetus depends on maternal TH until delivery [19]. The thyroid gland and its regulation by the HPT axis and negative feedback loops are summarized in Figure 2.

Pathophysiology of congenital hypothyroidism

There are two types of CH, permanent and transient, which in turn can be categorized into primary, secondary, and peripheral conditions [5]. Pathophysiologically, permanent CH can be divided into primary or central (secondary/tertiary) hypothyroidism [10]. Primary hypothyroidism is low TH levels in the blood due to thyroid gland damage [10]. Central hypothyroidism results from a TH deficiency due to pituitary or hypothalamic dysfunction [10]. Thyroid dysgenesis (TD) refers to a failure in thyroid gland formation, whereas dyshormonogenesis refers to a defect in TH production, both of which can highlight the primary CH [20]. TD accounts for most primary CH (80%) and includes a variety of anomalies such as agenesis, ectopic, or hypoplastic gland [21]. ​​The cause of TD is generally considered sporadic [22]. Research has suggested that genetic factors contribute to the disease [22]. According to recent findings, the development of the embryonic thyroid gland and its migration from the tongue base to the anterior neck is a multistage process involving highly regulated biochemical phases [23]. It requires the activation of transcription factors, such as thyroid transcription factor 1 (TTF-1), forkhead box protein E1 (FOXE1), NK2 homeobox 1 (NKX2-1), paired-box gene 8 (PAX-8), and transcription regulators (Table 2) [23]. The remaining 10-15% of primary CH are due to thyroid dyshormonogenesis [20]. Low T4 and T3 levels are standard in infants with primary CH, with high TSH and TRH levels due to a feedback signal to the hypothalamus and pituitary gland [16]. Peripheral hypothyroidism is caused by a dysfunction of TH transport, metabolism, or an under-response to TH [7]. Mutations can cause secondary congenital (central) hypothyroidism in the TSH or TRH receptor gene [21]. It is more commonly associated with congenital hypopituitarism, which may be caused by a mutation in a transcription factor gene that regulates pituitary development [21]. Central CH is rare, and infants will have low T4 or FT4, with low or low-normal TSH levels [16]. While these defects cause permanent CH, it is also possible for the condition to be transient CH [10]. The classical causes of transient CH are prematurity, maternal consumption of antithyroid drugs, maternal thyrotropin receptor-blocking antibodies, heterozygous mutations of DUOXA2 or THOX2, congenital hepatic hemangiomas, or maternal and neonatal iodine deficiency or excess [10]. It has been reported that antithyroid medications (methimazole, carbimazole, or propylthiouracil) taken by the mother may reduce the synthesis of TH in the newborn after birth, which can last for a few days to two weeks [7]. In neonates, maternal antibodies can cross the placenta and block TSH receptors, and this effect can last three to six months after birth as maternal antibody levels decline [7,24-25]. Iodine deficiency is common in European countries, specifically among premature infants, because maternal nutrition is deficient in iodine [7]. A high dose of iodine can cause CH in newborns, especially in preterm infants [26]. In addition, CH can also occur due to disorders affecting other organ systems, and these conditions are referred to as syndromic. Common forms of syndromic hypothyroidism include Bamforth-Lazarus syndrome, brain-lung-thyroid syndrome, and Pendred syndrome [7]. The clinical diseases of mutations in transcription factors associated with pediatric hypothyroidism are summarized in Table 1 [8].

**Table 1:** Clinical features of genetic conditions associated with congenital hypothyroidism FOXE1: forkhead box protein E1; NKX2-1: NK2 homeobox 1; NKX2-5: NK2 homeobox 5; PAX-8: paired-box gene 8; GLIS3: GLIS family zinc finger 3

Transcription Factors	Associated Malformation
FOXE1	Cleft palate, choanal atresia, spiky hair
NKX2-1	Neurological developmental delay and respiratory conditions
NKX2-5	Congenital heart conditions
PAX8	Urinary tract defects
GLIS3	Gastrointestinal, renal, endocrine conditions and development delay

Clinical aspects of congenital hypothyroidism

CH is the most prevalent congenital endocrine disorder and the most common preventable cause of intellectual disability worldwide [1]. Before the era of newborn screening programs, the incidence of CH was nearly 1 in 7,000 live births [27]. After establishing newborn screenings in the mid-1970s, the incidence has risen to 1 in 4,000 live births [28]. The incidence appears to increase over the past few decades due to the lowering of TSH screening cutoffs by newborn screening programs, which has increased finding for milder cases of CH [29]. With rising data from regional, provincial, and national screening programs, it is clear that the occurrence varies by region [7]. More than 95% of newborns with CH are rarely seen with clinical findings in the first several weeks due to protective effects provided by maternal TH passing through the placenta [30]. Since T4 has a seven-day half-life, maternal T4 can influence newborn infants three to four weeks after birth [30]. If hypothyroidism is left untreated or treated insufficiently, newborns with CH become symptomatic [31]. Early clinical manifestations of CH include hoarse cry, macroglossia, large fontanels, facial puffiness, lethargy, hypothermia, bradycardia, umbilical hernia, protuberant abdomen, feeding difficulties, hypotonia, constipation, and prolonged jaundice [31]. Additional clinical signs include generalized myxedema, poor growth, delayed deep tendon reflexes, developmental delay, and mental retardation if left untreated or undertreated [31]. Several studies in the USA have found that the Asian, Hispanic, and Native American populations had a higher incidence than the White population [7]. CH impacts the child's cognitive stages and neurological implications, regardless of whether the newborn has a mutated gene in the TH signaling structure, maternal hypothyroidism, or hormonally active agents that decrease TH usage [32]. The duration of fetal TH deficiency affects these outcomes differently [32]. The availability of maternal TH protects early brain development with CH, mainly through D2 (deiodinases)-mediated conversion of maternal FT4 [32]. As a result, regions of the brain that have just begun to develop, such as temporal and contextual memory, speech, hearing sorting, recognition, and executive handling, are impaired in CH [32]. When TH is insufficient in the first gestation, visual memory, gross motor development, sensory processing, and event imagination appear to be affected, comparable to maternal insufficiency [32].

Cretinism

Cretinism is characterized by severely stunted physical and mental development caused by untreated congenital TH deficiency [33]. Cretinism is always accompanied by significant cognitive dysfunction or hearing, speech, stance, gait, and growth defects [34]. There are two types of cretinism; symptoms of both types can sometimes be seen in the same individual [34]. Endemic neurological cretinism is distinguished by severe mental defect, eye strain, congenital deafness, and spastic diplegia in its matured form [33]. Almost the majority of the patients will have a goiter [33]. Mental deficiency is illustrated by a marked impairment of the capacity for abstract thinking, but vision is not affected [35]. Autonomic, vegetative, personal, and social functions and memory appear to be relatively well preserved except in the most severe cases [35]. The most striking characteristic is deafness, which may be complete in up to 50% of cretinism cases [35]. Almost all deaf cretinism patients are mute, and those with little hearing have no intelligible speech [35]. The rigidity of the upper and lower extremities and the trunk describes the movement disorder [35]. Proximal spasticity is present, with significantly exaggerated deep tendon reflexes in the knee, sternocleidomastoid, and biceps [35]. The leading cause of neurological cretinism is now thought to be maternal hypothyroidism caused by iodine deficiency [36]. On the other hand, Myxedematous cretinism is characterized by severe growth retardation, incomplete maturation of facial features, including the naso-orbital configuration, atrophy of the mandibles, puffy features, myxedematous, thickened and dry skin, dry and decreased hair, eyelashes, and brows, and significantly delayed sexual maturation [33]. Goiter is typically absent, and the thyroid is commonly not palpable, revealing thyroid atrophy [33].

Development of extrathyroidal congenital hypothyroidism

While cretinism remains a significant concern in iodine-deficient regions, TD results in the majority of cases of permanent CH in the iodine-sufficient areas, where developmental deviations occur during the embryonic life of the thyroid gland [5]. Even though most incidences of TD are infrequent, both environmental and hereditary components have been implicated in its underlying cause [5]. TD in newborns of some ethnicities and female versus male newborns and increased frequency of congenital deformities may have a genetic component [5]. Many patients have been identified as having transcription factor genetic defects [5]. Some of the transcription factors involved in thyroid gland development also participate in the development of other tissues such as the heart and kidneys [5]. In a population-based study conducted by Oliveri et al. in 2002 in Italy with 1420 newborns with CH (8.4%), TD was associated with increased extrathyroidal congenital abnormalities (Table 2) [37]. With a prevalence of 5.5%, heart abnormalities were the most common related malformations [37]. It is essential to understand that the developing heart and embryonic thyroid development are linked [38]. During the early stages of thyroid formation, the heart continues to descend, pulling the thyroid to its current position near the base of the neck [38]. Some congenital cardiac defects are related to developmental transcription factors [39]. In families with atrial septal abnormalities, tetralogy of Fallot, truncus arteriosus, aortic coarctation, double-outlet right ventricle, interrupted aortic arch, and L-transposition of the great arteries and the NKX2.5 gene have been discovered [40]. Similarly, another review of congenital heart abnormalities reported by Kresiener et al. in 2005 demonstrated that 76 infants with primary CH present malformation in 13.2% of cases, primarily cardiac involvement and cleft lips, cleft palate, and bifid spine (Table 2) [41]. Malformations were found in 10 (13.2%) of the 76 primary CH patients [41]. Eight out of 10 patients had isolated or combined cardiac malformations: atrial septal defect (n 2/10), ventricular septal defect (n 2/10), partial atrial-ventricular septal defect (n 2/10), valve anomalies (n 3/10), and other cardiac malformations (1/10) [41]. One patient had multiple malformations (ventricular septal defect and bifid spine) in addition to other abnormalities (neurogenic bladder and an extrapyramidal syndrome) [41]. 

Over the last decade, neonatal screening in children with CH has recognized a significant increase in extrathyroidal congenital malformation [42]. The most recent analysis evaluated the incidence of extra congenital abnormalities in a study of children with noted primary CH owing to TD [42]. In another study, 44 infants with major extra thyroid malformations were investigated by El Khloy et al. in Egypt in 2007 (Table 2) [42]. All anomalies were primarily affected by cardiovascular, urogenital, and musculoskeletal systems [42]. The increased risk of musculoskeletal abnormalities in this study was due to minor abnormalities such as brachydactyly (n = 9), thumb digitalization (n = 11), and cleft palate (n = 1) [42]. One patient had an absent left kidney and a right pelvic kidney while another had a missing left kidney [42]. In the USA, congenital abnormalities remain the leading cause of infant mortality. Children with CH have been reported to have defects in developing renal and urogenital systems [43]. However, no research has explicitly examined the prevalence of congenital renal and urologic defects in infants with CH [43]. In children, end-stage kidney disease is most commonly caused by congenital renal and urologic abnormalities, which account for about half of all [43]. Congenital renal and urologic abnormalities are not routinely screened in most infants with CH [43]. Mutations in the PAX 8 and FOXE1 genes have recently been linked to CH in patients with either isolated thyroid dysplasia or thyroid dysplasia with anomalies of the kidney, lung, forebrain, and palate [43]. A total of 980 newborns with CH found a strong link between renal and urinary tract abnormalities (Table 2) [43]. The other significantly increased defects in CH were cardiac, gastrointestinal, and skeletal [43]. Recent studies show that PAX 8 gene mutations are associated with CH in patients with thyroid dysplasia with related kidney abnormalities [44]. The PAX 8 gene works on the developing kidney's ureteric bud, mesonephric ducts, and primary collecting ducts [44]. Finally, in India, in 2010, Reddy et al. reported other thyroid abnormalities associated with increased spina bifida occulta, dysmorphic development, and congenital cardiac disease (Table 2) [45]. The most common associated anomalies, apparent exclusively on X-rays, were neural tube defects in the shape of spina bifida occulta, which was 41% [45]. All research shows that CH is part of a more significant developmental anomaly caused by hidden exposures during embryonic stages than a single event, based on the evidence [45].

**Table 2:** Prevalence of pediatric hypothyroidism and associated malformations USA: United States of America

Author and year of the study	Olivieri et al. (2002) [37]	Kresiener et al. (2005) [41]	El Khloy et al. (2007) [42]	Kumar et al. (2009) [43]	Reddy et al. (2010) [45]
Case series	1420	76	44	980	17
Population	Italy	Brazil	Egypt	USA	India
Congenital heart and great vessels anomalies	5.50%	10.50%	9.10%	17.30%	29.00%
Musculoskeletal anomalies	1.00%	-	47.70%	1.20%	-
Central nervous system	0.80%	-	-	-	41.00%
Digestive system	0.50%	-	-	2.90%	-
Urogenital malformations	0.40%	-	9.10%	8.70%	-
Cleft palate & lip	0.40%	-	-	-	-
Eye anomalies	-	-	4.50%	-	-
Total prevalence	8.40%	13.20%	70.50%	30.70%	59.00%

Diagnosis and management of congenital hypothyroidism

Any abnormal findings on newborn screening should be confirmed by measuring serum FT4 and TSH concentrations as soon as possible (preferably within 24 hours) [10]. All data must be evaluated using gestation and postpartum age reference varies as some specific labs could use FT4 and TSH reference ranges that aren't suitable for all infants [46]. Primary hypothyroidism affects infants whose TSH levels are elevated during newborn screening, and the intensity of the hypothyroidism necessitates immediate treatment [10]. Assume a patient's TSH level is higher than 40 mIU/L [47]. In that case, severe hypothyroidism must be considered, and therapy must begin immediately after confirmation blood lab tests are completed [47]. Even before conclusive tests are obtained, patients with serum TSH of more than 20 mIU/L or a reduced FT4 result despite TSH value should start treatment [47]. Additional testing in infants with primary CH may aid in identifying the etiology, thereby improving diagnosis and therapy plan [10]. Thyroid scans (either scintigraphy or sonography) could identify TD, an irreversible condition that needs medical attention even if the illness is minor [10]. On the other hand, an ectopic thyroid gland suggests the possibility of transient hypothyroidism [48]. Measurement of serum TG levels and radiographic examinations can provide important information concerning the pathogenesis of CH [49]. In recent studies, thorough updates in newborn screening have resulted in outstanding neurodevelopment results through efficient hormonal treatments, avoiding the uncertainty of intellectual impairment associated with cretinism [32].

It is not necessary to determine the underlying etiology when starting hormone therapy. Levothyroxine (L-T4) is the treatment of choice for CH [50]. Even though T3 is the organically dynamic structure of the hormone, T4 deiodination produces the majority of T3 in the brain. As a result, T3 alternate isn't required for optimal neurologic function [7]. Serum T3 stabilized and stayed stable regardless of the treatment dosage administered in research of 47 newborns given variable L-T4 treatment doses, demonstrating that treatment with only L-T4 is therapeutic [51]. A dose of 10 to 15 μg/kg/day is recommended by the American Academy of Pediatrics (APP) and can be adjusted based on the severity of hypothyroidism [52]. If the total daily prescription falls between 37.5 μg/kg/day and 50 μg/kg/day, an increased dose is advised, specifically during the first several weeks of treatment, to reduce the length of low FT4 levels [53]. According to the European Society for Pediatric Endocrinology (ESPE), L-T4 therapy must begin immediately, not longer than the first two weeks of birth, or as short as confirmative serum test results in newborns noted in a second screening program, to a beginning dose of 10 to 15 μg/kg/day [7]. A randomized controlled trial found that a higher amount causes THs levels to normalize faster and improves global intelligence quotient (IQ) but not verbal or performance IQ [51]. Longitudinal research on early and high dose therapy showed positive motor skills effects [54]. A lower initial dose and a diagnosis of athyreosis were associated with a lower IQ, yet the initial large amount did not enhance the overall result [32]. On the other hand, higher L-T4 doses have been linked to overtreatment, which can lead to poor outcomes [55]. Doctors are now treating the patients for three years to prevent impaired cognitive development and other illnesses like developmental delays [32]. Nevertheless, in the event of critical CH caused by athyreosis, perhaps an available treatment strategy may not show that it is possible to restore the intrauterine brain damage [32]. It is advisable to combine the crushed tablets with breast milk, formula milk, or water [53]. Liquid formulations of L-T4 have just been launched on the Italian market [56]. They offer advantages in terms of compliance since they are easier to administer to newborns and infants than tablets, which must be crushed and dissolved before administration [56]. The current study showed that both L-T4 formulations (liquid and tablet) normalized thyroid function within seven to 10 days of the administration, at a dosing range between 10 and 15 μg/kg/day, which agrees with the APP and ESPE principle goal for treating CH [56]. Soy-based dairy should be avoided because it can interfere with medication absorption [53]. A study from the United Kingdom shows that the formulation of infant "colic" (simethicone) drops inhibits LT-4 absorption [57]. It is essential to administer medication at the same time [52]. The efficacy of treatment and dosage should be assessed in the laboratory regularly [52]. The AAP recommends testing at two and four weeks after starting L-T4 treatment and more often if compliance is questioned or abnormal results are obtained [7].

Limitations

This article does not cover all the etiologies of CH, which are mild complications and were not considered in this study. Also excluded are chromosomal anomalies such as Down syndrome and Turner syndrome. It focuses on the pathophysiology, clinical aspects of congenital extra thyroid malformations, and treatment for the prevention of long-term complications, but it does not entirely address information from all the countries. Therefore, all relevant data related to congenital anomalies are not evaluated.

## Conclusions

According to the literature reviewed in this article, CH is a common endocrine disease that may be associated with severe congenital anomalies. Early identification and treatment are essential to avoid long-term negative consequences and enhance better results. The gold standard for diagnosing CH is universal newborn screening, which has resulted in a significant reduction in severe mental retardation caused by this illness. Moreover, further genetic studies are needed to enhance our understanding of the pathophysiology of congenital malformations related to hypothyroidism. To ensure that all newborns are correctly diagnosed and treated, these limitations must be acknowledged and recurrent monitoring is essential in high-risk newborns. A thorough evaluation that includes a medical exam, lab report, and supplementary image analysis will disclose the fundamental etiology of CH, which will help in diagnosis and treatment plan options. Therapy with L-T4 must begin soon, particularly within the first two weeks after birth, and thyroid performance should be established and sustained adequately. Infants with CH have an excellent prognosis when properly managed; however, minor abnormalities may persist in patients with severe hypothyroidism. Multiple gaps in knowledge exist regarding how to help hypothyroidism in infants effectively, including identifying patients with congenital malformations for treatment and the potential benefit of combined L-T3/L-T4 therapy for patients with persistent symptoms. The life-long effects on growth and development and possible long-term cardiovascular and psychosocial health are considerable, emphasizing the importance of future pediatric research investigating these conflicted areas.
